# The Epidemiology of Respiratory Syncytial Virus and the Impact of the COVID-19 Pandemic in a Retrospective Evaluation

**DOI:** 10.3390/pathogens14040375

**Published:** 2025-04-11

**Authors:** Paolo Solidoro, Antonio Curtoni, Cristina Costa, Francesco Giuseppe De Rosa, Alessandro Bondi, Francesca Sidoti, Nour Shbaklo, Filippo Patrucco, Davide Favre, Elisa Zanotto, Silvia Corcione, Rocco Francesco Rinaldo

**Affiliations:** 1 Division of Respiratory Medicine, Cardiovascular and Thoracic Department, AOU Città Della Salute e Della Scienza di Torino, University of Turin, 10126 Torino, Italy; paolo.solidoro@unito.it (P.S.); davide.favre@gmail.com (D.F.); roccofrancesco.rinaldo@unito.it (R.F.R.); 2 Medical Sciences Department, University of Turin, 10126 Torino, Italy; 3 Division of Virology, Department of Public Health and Pediatrics, AOU Città Della Salute e Della Scienza di Torino, University of Turin, 10126 Torino, Italy; acurtoni@cittadellasalute.to.it (A.C.); alessandro.bondi@unito.it (A.B.); francesca.sidoti@unito.it (F.S.); elisa.zanotto@unito.it (E.Z.); 4 Department of Medical Sciences, Unit of Infectious Diseases, University of Turin, 10149 Torino, Italy; francescogiuseppe.derosa@unito.it (F.G.D.R.); nour.shbaklo@unito.it (N.S.); silvia.corcione@unito.it (S.C.); 5 Respiratory Diseases Unit, Medical Department, AOU Maggiore Della Carità di Novara, 28100 Novara, Italy; filippo.patrucco@gmail.com; 6 Division of Geographic Medicine, Tufts University School of Medicine, Tufts University, Boston, MA 02111, USA

**Keywords:** respiratory syncytial virus, COVID-19, epidemiology, pandemic

## Abstract

Introduction: Respiratory syncytial virus (RSV) is the main etiological agent in pediatric lower respiratory tract infections. The limited availability of therapeutic options for severe clinical cases associated with RSV infection makes prophylactic interventions a priority for containment. The aim of the current study was to evaluate the epidemiology of RSV in the Piedmont population and the consequences of containment measures applied during the pandemic on viral circulation in the immediate and medium-term post-pandemic phase. Methods: This study considered all biological samples analyzed for RSV at the City of Health and Science of Turin collected from 1 January 2016 to 31 December 2023. Evaluation of the positivity rates of samples was performed and differences between pediatric and adult population swabs (nasopharyngeal, pharyngeal, nasal aspirates) and bronchoalveolar samples were reported. Results: This study analyzed 14,085 samples and highlighted a trend in Piedmont RSV infections characterized by a higher pediatric population involvement of 82% compared to the adult population at 17%. A higher number of URT infections (95%) compared to LRT infections (4.6%) was also identified. This study shows a peak in RSV cases from November to April between 2016 and 2020. Our data show no RSV positivity during the 2020/2021 winter season, a result most likely due to the influence of containment measures implemented during the COVID-19 pandemic. Conclusions: Our study provided an epidemiological panorama of RSV and its high prevalence in pediatrics and adults. Pediatrics had a higher prevalence, while adults presented a delayed trend of about one month compared to pediatrics. The effectiveness of infection control measures applied during the SARS-CoV-2 pandemic to limit viral infections were proved. Future studies may further investigate the impact of the SARS pandemic on RSV epidemiology considering patients at a higher risk of severe symptoms.

## 1. Introduction

Respiratory syncytial virus (RSV) is the main causative agent of acute respiratory tract infections (RTIs) in pediatrics, with cases estimated at 30 million and 100,000 deaths per year. In pediatric subjects, the most significant clinical manifestations are bronchiolitis, characterized by respiratory distress, cough, hypoxia, and apnea [1]. RSV is also a cause of RTI in adults and elders, with a global rank second to influenza among respiratory viruses (1–10% of cases) [2]. Pneumonia symptoms are seen in patients with broncho pneumopathy, chronic obstructive pulmonary disease (COPD), and acute bronchitis. However, RSV in immunocompetent adults is usually characterized by mild symptoms but still acts as an important reservoir that facilitates transmissibility and diffusion [3].

RSV is characterized by high contagiousness, having on average an R_0_ of 3. Until the SARS-CoV-2 pandemic, RSV had a seasonal pattern characterized by a peak in winter months [3]. However, several studies have observed a change in the spread of the virus after the pandemic, showing an increase in RSV infections outside the winter period [4,5,6].

During the pandemic, it was indirectly observed how the isolation of symptomatic subjects, triage, reduced mobility, and a decrease in social interactions imposed by the lockdown led to a drastic drop not only in COVID-19 cases but also in other respiratory viruses including RSV. Nevertheless, with the easing of restrictions, a return of RSV cases was seen, although cases had atypical features, as they manifested outside the normal seasonal trend and affected generally component age groups such as young adults and non-neonates [4,5,6,7,8].

Epidemiological data focusing on specific geographic regions, such as Piedmont, Italy, are lacking. Most existing research tends to aggregate data at a broader level, which may overlook regional variations in RSV incidence, seasonal patterns, and demographic factors affecting transmission. While some studies have explored how COVID-19 containment measures have influenced the transmission of respiratory viruses, there is limited data on the specific effects of these measures on RSV dynamics. A detailed examination of RSV trends before, during, and after the COVID-19 pandemic is necessary to understand the full impact of the pandemic on RSV epidemiology.

This study aims to fill the gaps by providing a comprehensive analysis of RSV epidemiology in the Piedmont region from January 2016 to December 2023. We specifically investigated the trends in RSV infections before, during, and after the COVID-19 pandemic, highlighting the implications of containment measures on RSV circulation patterns. By elucidating these dynamics, our findings contributed to a more nuanced understanding of RSV behavior during and post pandemic, ultimately informing public health policies and vaccination strategies tailored to mitigate the impact of RSV in vulnerable populations.

## 2. Materials and Methods

This study was conducted at the City of Health and Science of Turin, the largest tertiary care center in north-west Italy. All samples tested for RSV, regardless the reason of testing, were collected from 1 January 2016 to 31 December 2023. Samples were categorized per age (pediatric or adult) and specimen (swabs or bronchoalveolar sample).

The aim of this study was to define the rates of RSV in the pediatric and adult population in the studied setting. In addition, we evaluated the impact of the SARS-CoV-2 pandemic and the preventive measures applied in 2020–2021 on the epidemiology of RSV infection. We also highlighted the consequences that the SARS-CoV-2 pandemic had on RSV cases in the subsequent period, namely from June 2021 until the end of 2023.

### 2.1. Comparison of Seasonal Peaks

The study focused on evaluating the possible presence of alterations between the seasonal peaks, particularly by comparing the years before the pandemic with the years after. Initially, a graph was created with the annual positivity rates, defined as the number of samples that tested positive per the total number of samples recorded annually.

However, this approach was not proven effective in providing a comparison between the seasonal peaks as the annual evaluation would have divided the samples of the same peak into two different years. This is because the seasonal peak is mainly localized in the winter period extending over two consecutive years.

Hence, we analyzed the samples in reference to a different time interval, between 1st July of the year before and 30 June of the year after. This process was repeated for all the years included in the study, up to 2022–2023. In this way, it was possible to include all the samples belonging to the same peak in a single time interval and therefore compare seasonal positivity rates.

### 2.2. Microbiology Processing

Two types of molecular panels were used to analyze the samples: GeneXpert Cepheid Xpert Xpress^®^ Flu/RSV and Filmarray Respiratory Panel. GeneXpert Cepheid Xpert Xpress^®^ Flu/RSV is a CE-IVD-certified test, with a specificity and sensitivity higher than 98% for RSV and influenza A and B. In most cases, the GeneXpert Cepheid was used as the first test as it identifies the main pathogens capable of causing severe respiratory infections. In case of negativity, further analysis was carried by Filmarray to extend the search. However, when rapid diagnosis was necessary for certain cases, Filmarray was used in the first place.

Identifiable pathogens from this panel are adenovirus, coronavirus 229E, HKU1, OC43 and NL63, Human Metapneumovirus, Human Rhinovirus/Enterovirus, influenza A, A/H1, A/H1-2009, A/H3, B, Parainfluenza 1-4, RSV, *Bordetella pertussis*, *Chlamydophila pneumoniae*, and *Mycoplasma pneumoniae*.

### 2.3. Statistical Analysis

The results were expressed as absolute numbers and percentages. Monthly positivity rates and seasonal trends were expressed as the ratio of positive samples to the total samples produced in that given month considering all years, from 2016 to 2023, and then expressed as a percentage. Chi-square tests were performed to determine the significance of differences observed in RSV-positive and -negative groups. These tests were performed at a significance level of *p* < 0.05 using SPSS version 24.

## 3. Results

In total, 14,085 samples were evaluated in this study, of which 1608 (11%) were positive for RSV. Pediatrics accounted for N = 8791 (62%) and N = 5197 (36%) were adults, as reported in Table 1. We excluded 231 (1.62%) “undefined” samples from further analysis as it has not been possible to classify them as a pediatric or adult. Table 2 and Table 3 describe the distribution in adult and pediatric patients, respectively.

Of the total of specimens collected, 12,272 (87%) swabs were reported. Nasal swabs/aspirates accounted for the majority (N = 7176—51%), followed by 2488 (17%) being nasopharyngeal swabs and 2608 (18%) being pharyngeal swabs. Additionally, bronchoalveolar samples accounted for 12% of the total, comprising 10.5% bronchoalveolar lavages (1494 samples), 2.1% broncho aspirates (305 samples), and 0.07% respiratory biopsies (10 samples).

### 3.1. Overall Positive Rates

The study results show a positive rate of approximately 11% of the specimens. A total of 1534 swabs (12.5% of the total number of swabs) and 74 bronchoalveolar samples (4% of the total number of samples) were positive. A higher positivity rate was observed in the pediatrics than in the adults. Regarding specimen type, the highest number of positive specimens were swabs (1534, 94%) compared to bronchoalveolar samples (74, 4.5%). Of note, 60% of the swabs were from pediatric departments (N = 8791) and only 36% from adults (N = 5197). In contrary, bronchoalveolar specimens accounted for 76% (N = 1387) in adults and 19.4% in pediatrics (N = 351).

Stratifying the swabs, a higher positivity rate (28%) was seen in nasopharyngeal swabs, versus pharyngeal and nasal aspirates (16% and 49%, respectively). In the bronchoalveolar samples, a higher positivity rate was observed in BAL (2.7%) versus broncho aspirates 1.8%. Broncho aspirates were performed mainly in the pediatric population (76%), while BAL was mainly in adults (88%).

### 3.2. Trend in Cases During 2016–2023

**Figure 1:**
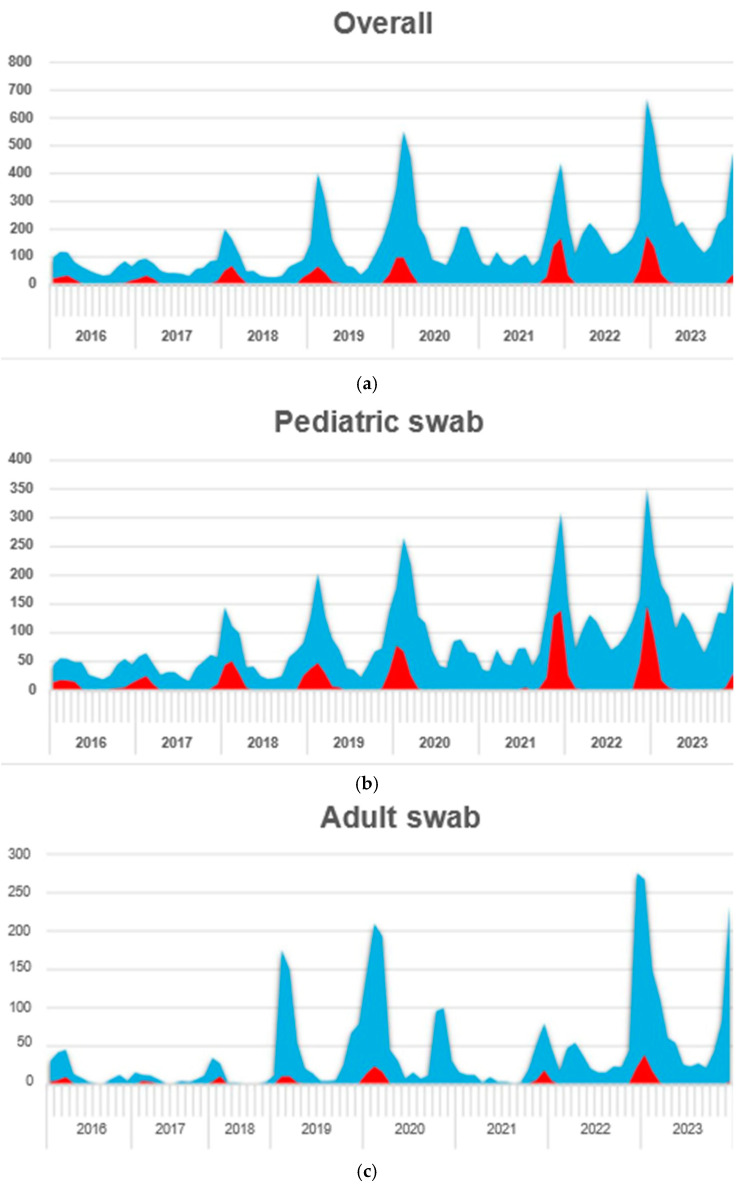
Swab trends: (**a**) overall, (**b**) pediatric, and (**c**) adults. (x-axis: years; y-axis: frequency; red area: positive cases; blue area: negative cases).

Figure 1 shows how requests for analysis for respiratory viruses were increasing in the same period in which RSV positivity occurred, with a prevalence in winter months. Furthermore, there seemed to be a progressive increase in cases over the years, except for winter 2020–2021 where no cases were found. Evaluating pediatric swabs, we generally observed a higher rate of positive swabs compared to the adult population.

Figure 1 also shows the increased number of cases after the pandemic compared to the previous years. In adults, we observed less positivity even during the winter period. The increase was also clear in the number of samples collected in adults in 2019 compared to previous years. The increase in adults was less evident following the pandemic compared to what was observed in pediatric swabs.

### 3.3. Monthly Positive Rates and Seasonal Trends

**Figure 2:**
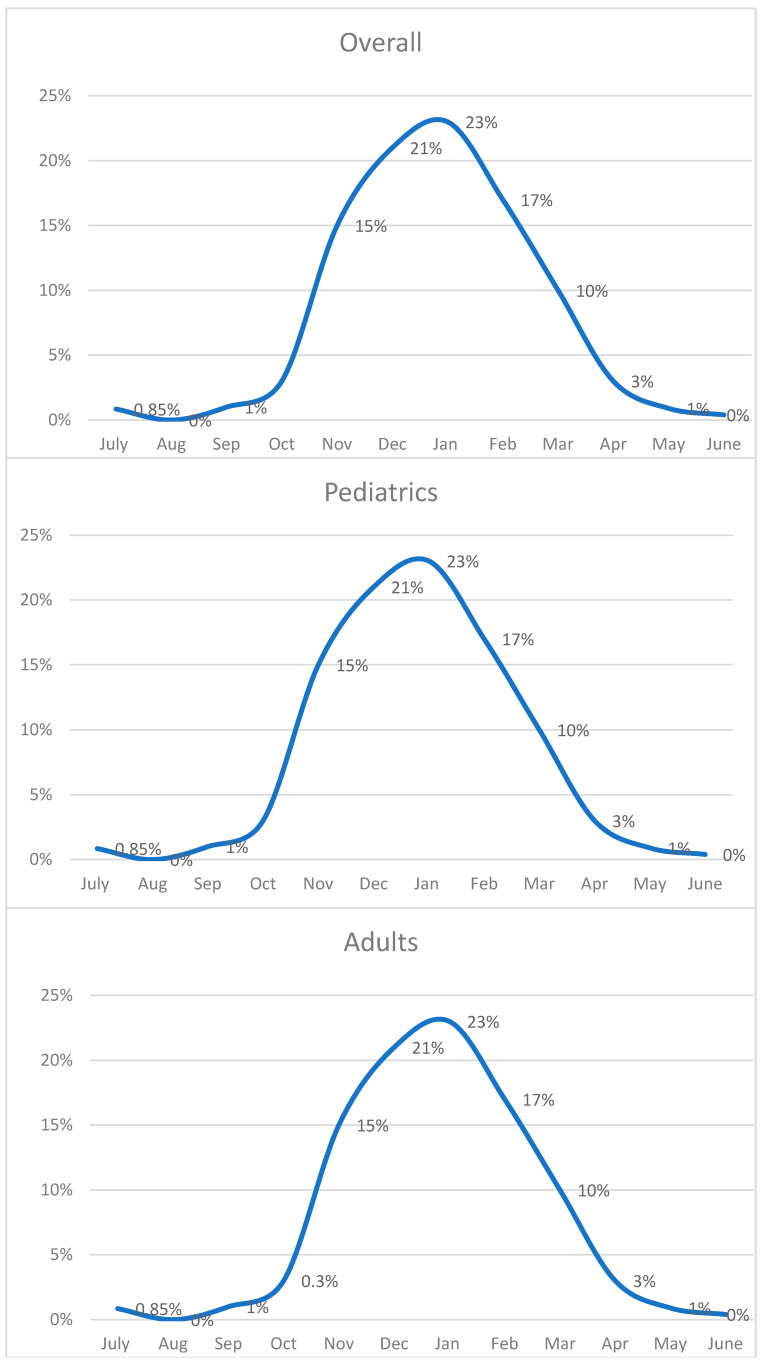
Positive rates per month (2016–2023) (x-axis: months; y-axis: positive rates (%)).

The monthly positivity rate of the samples was also evaluated to verify the seasonality of the virus. Figure 2 shows how the overall RSV-positive cases were increasing in October, peaking between December and January, and decreasing progressively until April.

In pediatrics, the curve was like the overall curve, with the peak in the majority of cases being in agreement. In the months of greatest interest, the positive rate reached above 20%. In adults, the cases began to increase in November, peaking between January and February, and gradually decreasing until May. In this case, the maximum positive rate was around 10%.

### 3.4. Comparison of Seasonal Peaks Results

**Figure 3:**
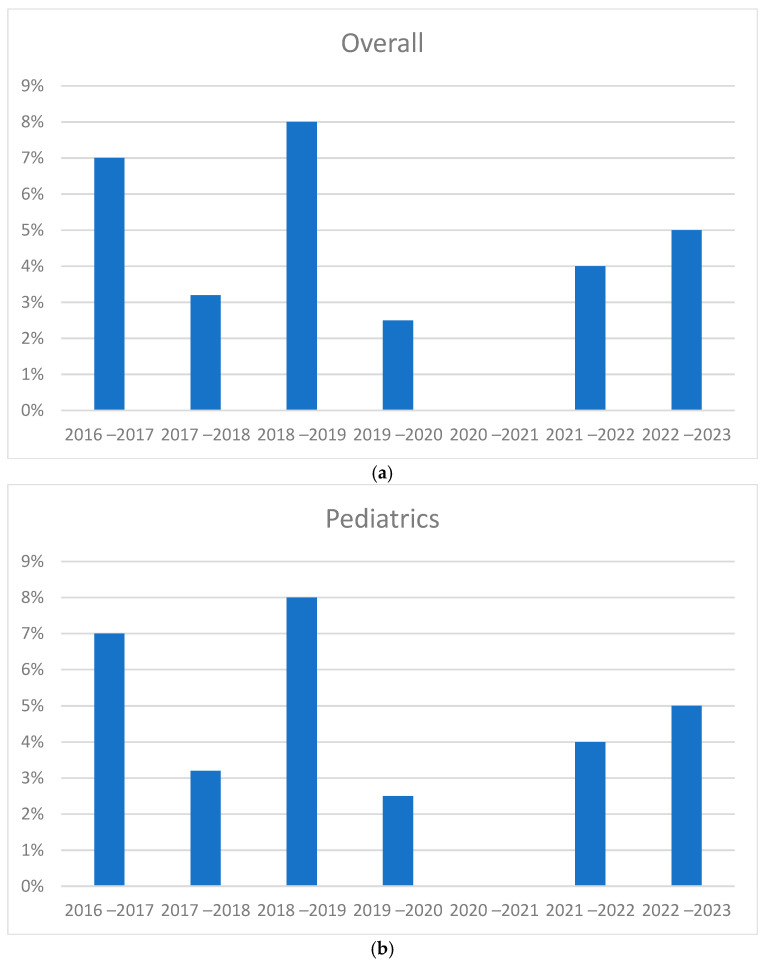

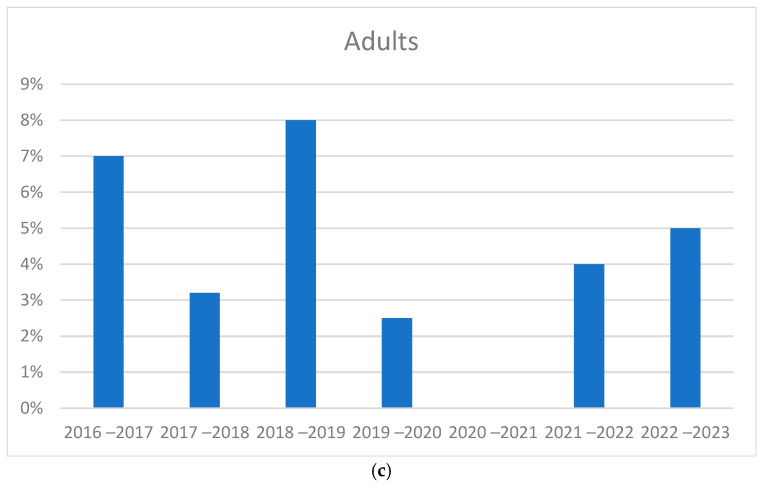
Absolute numbers of RSV-positive samples by seasonal peak—(**a**) overall, (**b**) pediatric, and (**c**) adult (x-axis: year; y-axis: frequency).

An evaluation for absolute numbers was carried out to check for any variations in the years (Figure 3). The growth trend was increasing before the pandemic. Starting from the positivity rate in pediatrics, no significant increase or difference was observed in the post-pandemic period. While considering the absolute numbers in pediatrics, there was an increase in cases in seasonal peaks during 2021–2022 and 2022–2023 compared to previous years. In adults, an increase in positivity rates was not seen in the post-pandemic period. In terms of absolute numbers, an increase in cases was found in 2022–2023, while in the previous year positivity was found to be like the pre-pandemic period.

**Figure 4:**
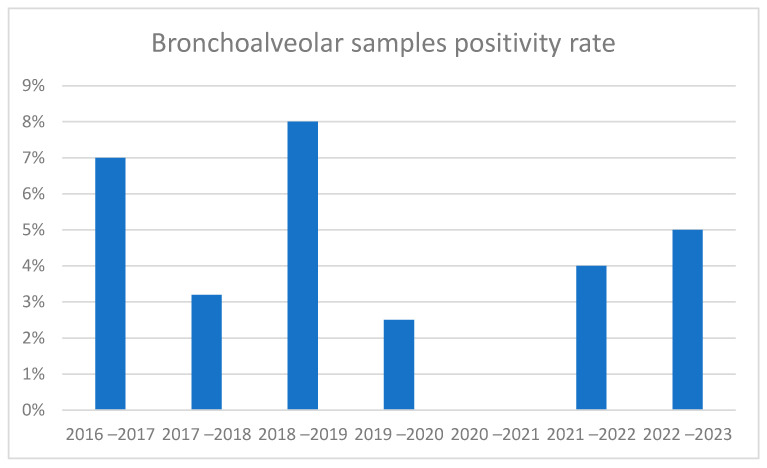
Positivity rate of bronchoalveolar samples by seasonal peak (x-axis: years; y-axis: percentage).

In bronchoalveolar samples, positivity rates were found to be higher in the pre-pandemic period than in the post-pandemic period (Figure 4). Meanwhile, in the analysis of absolute values, more cases were found in the periods following the pandemic. It should be considered that in this case, the psitive samples are much fewer than the swabs (about ten per year); therefore, the variations between the epidemic peaks are scarcely relevant.

Figure 4 shows the absence of cases in the period 2020–2021. The seasonal peak immediately following the pandemic in 2021–2022 is clearly shifted to the left. In 2021–2022, cases began to be recorded as early as the summer period (August–September), reached their peak in November and December, and resolved in February. In the years before the pandemic, however, cases began to be recorded in November, with the peak seen in the months of January or February and with slight differences from year to year, continuing until April. Regarding 2022–2023, there was an intermediate trend, with the start of cases in the period of October, the peak in December, and the exhaustion of cases in March.

**Figure 5:**
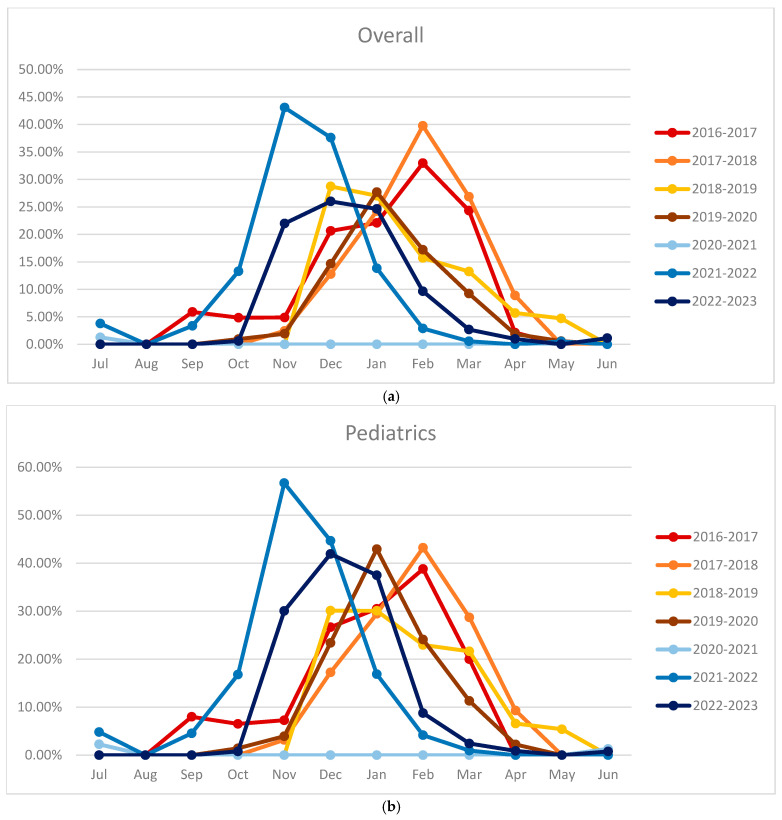

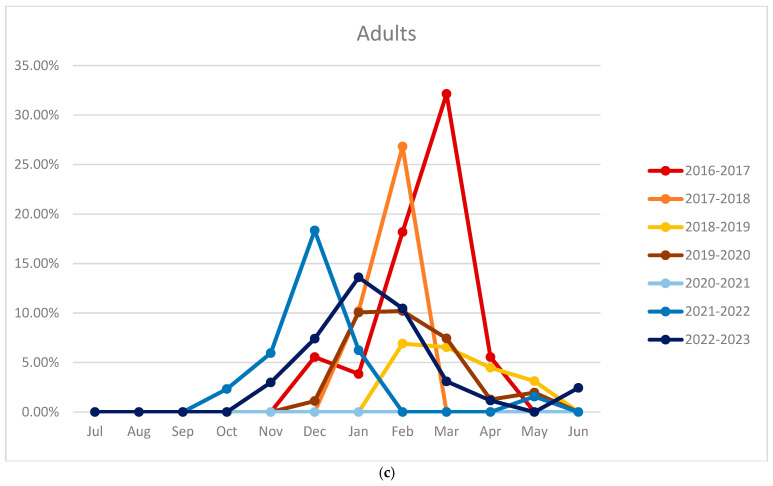
Comparison between the seasonal peaks—(**a**) overall, (**b**) pediatric, and (**c**) adult (x-axis: months; y-axis: percentage).

In pediatrics, the peak of 2021–2022 occurred before the typical months in which RSV cases usually occurred, while the rates for the year 2022–2023 follow an intermediate trend. Regarding adults, a trend was observed between the various seasonal peaks but shifted one month further than the pediatric population, as already observed in the monthly trend. In this case, the peak of 2021–2022 was indeed anticipated compared to the following years. However, compared with the pediatric population, the peak was in December rather than in November for adults and pediatrics, respectively. Similarly, cases recorded before the pandemic peaked during February and March rather than January and February for adults and pediatrics, respectively. Finally, the cases of 2022–2023 appear to again follow an intermediate trend between that observed in the previous period to the pandemic and the one immediately after. Figure 5 shows the percentages of seasonal peaks.

## 4. Discussion

We studied the epidemiology of RSV in the whole population and categorized cases into pediatrics and adults and according to the type of samples. This study highlights how the epidemiology of RSV in the Piedmont territory is largely superimposable to what was found in other studies conducted in developed countries with temperate climates such as Germany or the USA.

Our study found an overall positivity rate of the samples equal to 11.4%, but when analyzing the trend over time, the cases were mainly recorded in winter. In fact, the rate of monthly positivity increased significantly in these periods, reaching 25–40% of cases. These values are in parallel with the literature [9,10], as they respect the known trend of RSV cases during the year, especially for the data recorded in the seasonal peaks that extend from 2016 to 2020.

Our study highlights the importance of integrating RSV surveillance into broader respiratory virus monitoring systems. The significant shifts in RSV patterns observed during and after the pandemic underscore the need for adaptable public health responses that can quickly address emerging trends in respiratory infections. This includes the need for timely communication, resource allocation, and community engagement in vaccination campaigns to ensure high coverage rates.

Regarding our RSV sampling strategy, no change in sample collection was applied during the studied periods. The same procedure was performed during the COVID-19 pandemic as well without specific imposition. Our standard RSV screening was based on clinical and radiological criteria to identify a suspected interstitial viral infection (for example transplanted patient with symptoms). It is possible that during the COVID-19 pandemic, the pool of patients tested was different, including more acute patients suspected for viral pneumoniae and immunocompromised or onco-hematological patients whom were not able to access the hospital.

Classifying the samples by age and specimen type, positivity rates were different. Positivity rates were higher in the pediatrics than in the adults, which is in line with the literature where a greater tropism of the virus is highlighted in children, leading to a more severe clinical picture and requirement for rapid diagnosis. In fact, the overall monthly positivity rate in the pediatric population reached the maximum value of 30% in the months of December and January versus around 10% in January and February for adults.

Another difference between the two populations was the delay in the seasonal trend in adults by about one month compared to pediatrics. This phenomenon could be due to the virus circulating first among pediatrics who are at greater risk of contracting the infection.

We divided the specimens into swabs and bronchoalveolar samples because invasive diagnostic methods of the lower respiratory tract, such as bronchoalveolar lavage, are rarely used in pediatrics and are more limited to adults.

Another aspect that we observed was the trend of the samples analyzed over time. The number of samples analyzed increased over the years. However, this trend showed an important decline during the pandemic, followed by a rapid recovery. Despite the increased number of samples analyzed, the seasonal positivity rate remained generally unchanged (12–16%), except for the seasonal peak of 2020–2021 during which no cases were recorded. These data agree with the study conducted by Rios-Guzman et al. [5] which also found an increase in the number of swabs analyzed over the years, associated with a rate of constant positivity with seasonal values between 15 and 20%.

In adults and in bronchoalveolar samples, the positivity rate was higher in the first years studied (2016–2018). However, evaluating the cases in the absolute number, the lowest number of positives was found in these years. This could be justified by the fact that in the first years considered in this study, this type of etiological investigation in adults was limited to very specific clinical cases. Over time, diagnosis has become more accessible and was extended to other patients, increasing the overall RSV cases detected but reducing the positivity rate.

Overall, excluding the winter period 2020–2021, there was an increasing trend in RSV cases in absolute numbers, especially in the pediatric population. In particular, pediatrics had a significant increase in cases in the year following the pandemic (2021–2022). On the other hand, adults had a significant increase only in the winter of 2022–2023. This could be because part of the restrictions were still implemented during the pandemic period and consequently limited the spread in adults.

### Effect of Infection Control Measures Applied During SARS-CoV-2 Pandemic on RSV

Several studies [6,7] have highlighted how infection control measures are able to counteract the spread of most respiratory viruses, including RSV, as they act on the transmission routes [4,5,11,12,13,14,15]. During the restrictions applied in the winter of 2020–2021, RSV cases were drastically reduced if not eliminated. Similarly, in our study, no RSV cases were recorded during this period, neither in the pediatrics nor adults.

The last aspect considered in this study was to investigate whether and how the pandemic from SARS-CoV-2 had altered the epidemiology of RSV in the following years, given that many studies in the literature have found changes from the known seasonality trend in cases in the post-pandemic period [16,17,18,19]. Our results are in line with other studies that evaluated the epidemiology of the virus in the period following to the pandemic [4,5,20,21]. Interestingly, the pandemic not only caused an increase in RSV cases in the subsequent years but also changed the seasonal trend of the virus. Thus, the seasonal peak of RSV was brought forward by two months in the period immediately following the pandemic compared to the known spread of the virus.

The phenomenon of increase in positive samples and the anticipation in autumn or even summer months in the period following the pandemic has also been reported by other studies. In fact, the study by Abu-Raya et al. [22] evaluated the reasons behind this phenomenon. The most accredited theory seemed to be that of “immune debt”. Immune debt is defined as a lack of exposure to the virus during the lockdown of winter 2020–2021, resulting in a decrease in immunized subjects, especially pediatrics who contract RSV during their first year of life [23,24]. Once the restrictions were eased, the virus started circulating again but in a population significantly more sensitive than in the pre-pandemic period, causing a more rapid and aggressive spread. The lack of immunization led to the formation of a pool of subjects sensitive to the virus, facilitating rapid circulation once the pandemic restrictions were removed. The same concept also applies to adults, though in a less striking way, where the real increase in absolute numbers was observed in 2022–2023 and not immediately after the pandemic, unlike to what has been reported in pediatrics.

Given the potential for altered seasonal patterns and increased susceptibility in populations due to “immune debt”, our results reveal the importance of evaluating and possibly revising vaccination strategies for RSV. This could include advocating the development and distribution of RSV vaccines, particularly targeting vulnerable populations such as infants and the elderly, to enhance immunity and reduce the burden of disease.

Future research should assess cyclic patterns and self-correlation in RSV positivity rates over time. Moreover, the addition of demographics such as age distribution, clinical data, and sub-classification per different specimen types to future research shall enhance our understanding of how various factors influence RSV transmission and infection patterns.

A limitation of our study includes not focusing on RSV-positive patients but rather on the number of samples analyzed. In fact, some patients may have repeated the microbiological tests several times, especially if the first samples were negative. For this reason, this study may have underestimated the actual impact of RSV, especially during seasonal peaks. However, the complete absence of cases in summer periods or during the winter of 2020–2021 confirms the period in which the RSV was circulating and when it was not expected to be.

Another limitation is the absence of the date of birth of the patients, as well as the demographic or clinical data for the patients who gave samples. These data are important as they allow us to stratify further the categories of patients by identifying subjects most at risk. For example, in the pediatric population, they are at a greater risk in early childhood compared to in the second or third grade or to adolescents. Similarly, there is also variation in the adult population risk based on age; in fact, in the literature, it emerges that elderly subjects are at a greater risk of having serious RSV infections compared to young adults.

Another limitation is the absence of clinical information to categorize the severity of the patients and consequent comorbidities that increase RSV risk. Data were available on the department of admission only.

## 5. Conclusions

Our study confirmed the high diffusion of RSV in pediatrics and adults, highlighting the differences between the two groups. The pediatric population had a higher prevalence, while adults presented a delayed trend of about one month compared to pediatrics. This study also proved the effectiveness of infection control measures applied during the SARS-CoV-2 pandemic and how the post-pandemic period was characterized by a greater number of cases, especially in the pediatric population, with an onset of two months earlier than the usual seasonal peak. The trend after the pandemic showed a partial return to the previous seasonal trend, while the increase in cases continued. Future studies may further investigate the impact of the SARS pandemic on RSV epidemiology considering patients at a higher risk of severe symptoms.

In conclusion, the current study gives an epidemiological panorama of RSV over a considerable period. The description of epidemiology and seasonal peaks highlights the necessity of creating a preventive strategy that is applicable in the long-term reasonably combining vaccinations and infection control.

## Figures and Tables

**Table 1:** Overall descriptive distribution of collected samples.

Variable	Negative N = 12,477 (88.5%)	Positive N = 1608 (11.5)	Total N = 14,085 (100)	*p*-Value
Gender				
Female	5589 (44.7)	737 (45.8)	6326 (44)	0.627
Male	7014 (56.2)	879 (54.6)	7893 (56)	0.395
Ward				
Adult	4927 (39.4)	270 (16.7)	5197 (36)	<0.001
Undefined	216 (1.7)	15 (0.93)	231 (1.6)	0.019
Pediatric	7460 (59.7)	1331 (82.7)	8791 (62.4)	<0.001
Material type				
A. Bronchoalveolar sample	1739 (13.9)	74 (4.6)	1813 (12.8)	<0.001
Biopsy/excision	3 (0.02)		3 (0.02)	-
Respiratory biopsy	10 (0.08)		10 (0.07)	-
Broncho aspirate	275 (2.2)	30 (1.8)	305 (2.16)	0.389
Bronchoalveolar lavage	1450 (11.6)	44 (2.7)	1494 (10.6)	<0.001
B. Swabs	10,738 (86)	1534 (95.3)	12,272 (87)	0.006
Pharyngeal	2341(18.7)	267 (16.6)	2608 (18.5)	0.079
Nasopharyngeal	2027 (16.2)	461 (28.6)	2488 (17.6)	<0.001
Nasal swab/aspirate	6370 (51)	806 (50)	7176 (50.9)	0.688
Positive tests per specimen group				
Swab	10,738 (87.5)	1534 (12.5)	12,272 (100)	0.006
Broncoalveolar	1739 (96)	74 (4)	1813 (100)	<0.001

**Table 2:** Descriptive data of adults.

Variable	Negative N = 4927 (94)	Positive N = 270 (5.1)	Total N = 5197 (100)	*p*-Value
Gender				
Female	2116 (43)	115 (42)	2231 (43)	0.908
Male	2811 (57)	155 (57)	2966 (57)	
Material type				
A. Bronchoalveolar sample	1349 (27)	38 (14)	1387 (26)	<0.001
Biopsy/excision	3 (0.06)		3 (0.05)	
Respiratory biopsy	9 (0.18)		9 (0.17)	
Broncho aspirate	59 (1.19)	1 (0.3)	60 (1.15)	0.219
Bronchoalveolar lavage	1278 (26)	37 (13.7)	1315 (25)	<0.001
B. Swabs	3487 (70)	230 (85)	3717 (71)	0.044
Pharyngeal	1180 (24)	69 (25)	1249 (24)	0.639
Nasopharyngeal	411 (8.3)	26 (9.6)	437 (8.4)	<0.001
Nasal swab/aspirate	1896 (38.4)	135 (50)	2031 (39)	0.015
Positive tests per specimen group				
Swab	3487 (93)	230 (6.19)	3717 (100)	
Broncoalveolar	1349 (97)	38 (2.74)	1387 (100)	

**Table 3:** Descriptive data of pediatrics.

Variable	Negative N = 7460	Positive N = 1330	Total N = 8791	*p*-Value
Gender				
Female	3385 (45)	618 (46)	4003 (45)	0.653
Male	4075 (54)	713 (53)	4788 (54)	0.709
Material type				
A. Bronchoalveolar sample	317 (4.2)	34 (2.5)	351 (3.9)	0.004
Broncho aspirate	206 (2.7)	27 (2)	206 (2.3)	<0.001
Bronchoalveolar lavage	110 (1.4)	7 (0.5)	110 (1.2)	0.005
B. Swabs	7114 (95)	1295 (97)	7114 (80)	0.623
Pharyngeal	1126 (15)	197 (14)	1126 (12)	0.819
Nasopharyngeal	1555 (20)	430 (32)	1555 (17)	<0.001
Nasal swab/aspirate	4433 (59)	668 (50)	4433 (50)	<0.001
Positive tests per specimen group				
Swab	7114 (84.6)	1295 (15.4)	8409 (100)	
Broncoalveolar	317 (90.3)	34 (9.6)	351 (100)	

## Data Availability

The original contributions presented in this study are included in the article. Further inquiries can be directed to the corresponding author.
